# Localization of the therapeutic targets for endothelin receptor antagonists and sodium-glucose co-transporter 2 inhibitors in the chronic liver disease, primary sclerosing cholangitis

**DOI:** 10.3389/fphar.2025.1680875

**Published:** 2025-09-29

**Authors:** Rhoda E. Kuc, Anna L. Paterson, Thomas L. Williams, William T. H. Gelson, Peter J. Greasley, Phil Ambery, Janet J. Maguire, Anthony P. Davenport

**Affiliations:** ^1^ Experimental Medicine and Immunotherapeutics, Addenbrooke’s Hospital, University of Cambridge, Cambridge, United Kingdom; ^2^ Department of Pathology, Cambridge University Hospitals NHS Foundation Trust, Cambridge, United Kingdom; ^3^ Department of Medicine, University of Cambridge, Cambridge, United Kingdom; ^4^ Cambridge Liver Unit, Cambridge University Hospitals NHS Foundation Trust, Cambridge, United Kingdom; ^5^ Early Clinical Development, Research and Early Development, Cardiovascular, Renal and Metabolism (CVRM), BioPharmaceuticals R&D, AstraZeneca, Gothenburg, Sweden; ^6^ Late-Stage Development, Cardiovascular, Renal and Metabolism, BioPharmaceuticals R&D, AstraZeneca, Gothenburg, Sweden

**Keywords:** endothelin-1, ETA, ETB, immunofluorescence, primary sclerosing cholangitis, sodium-glucose co-transporter-2, SGLT-2, solute carrier family 5 member 2

## Abstract

**Introduction:**

Primary sclerosing cholangitis (PSC) is a chronic liver disease of unknown cause contributing to cirrhosis and cancer but has no cure. PSC is characterized by inflammation within ductal fibrosis, progressive bile duct narrowing and loss, with damage to cholangiocytes (epithelial cells affecting bile production) and liver repair. ET-1, produced by cholangiocytes, contributes to fibrosis, vasoconstriction, and inflammation via ET_A_ receptors. In patients, ET-1 and ET_A_ gene expression are elevated and ET_A_ antagonists reduce disease progression in PSC animal models. Ongoing clinical trials of portal hypertension in liver disease are testing the efficacy of a new treatment strategy combining ET_A_-selective antagonist zibotentan with SGLT2 inhibitor dapagliflozin.

**Methods:**

To interrogate the potential of a comparable strategy in PSC we have initially compared the localization of ET receptors and SGLT2 transporter in human PSC liver.

**Results:**

In ethically sourced healthy human liver, ET_A_ immunofluorescence was primarily found in bile duct epithelial cells within the portal tract, smooth muscle of the central vein, with low levels in hepatocytes. SGLT2 immunofluorescence was mainly detected on bile duct epithelial cells and hepatocytes. ET_A_ co-localized with smooth muscle cells in large arteries and veins, while ET_B_ immunoreactivity was present in hepatocytes and endothelial cells. In the PSC vasculature, the pattern of expression of smooth muscle ET_A_ receptors that mediate vasoconstriction was retained, consistent with the hypothesis that ET_A_ selective antagonists would be beneficial in reducing portal hypertension. ET_B_ receptors were principally localised on endothelial cells and would be expected to mediate beneficial vasodilation. In diseased areas, all three proteins localised to ductal reactions, reflecting the response of the liver to injury, involving cholangiocyte proliferation, promoting beneficial regeneration but also associated with fibrosis and inflammation. Both ET_A,_ ET_B_ and low levels of SGLT2 immunofluorescence localised to fibroblasts within the fibrous septa where bands of scar tissue can restrict hepatic blood flow, leading to cirrhosis.

**Discussion:**

Both drug targets were retained in the key hallmarks of PSC pathology; ET_A_ and SGLT2 staining within cholangiocytes undergoing ductal transformation and cells within the fibrotic septa, supporting the proposed benefit of combination treatment strategy.

## 1 Introduction

Primary sclerosing cholangitis (PSC) is a chronic liver disease of unknown aetiology. PSC is associated with significant morbidity (such as cirrhosis and cancer) and mortality, with an estimated prevalence rate of ∼14 per 100,000 persons (Bowlus et al., 2023; Cooper et al., 2024). There is currently no cure for PSC and treatments are limited to managing symptoms and complications. These include ursodeoxycholic acid that improves liver function tests by increasing bile flow and preventing liver cell damage, but it does not significantly impact survival or the need for liver transplantation. Additionally, bile acid sequestrants are used to bind bile acids to relieve itch and antibiotics are used to treat infections caused by biliary obstruction (Tan et al., 2023; Curto et al., 2025). Interventional options include endoscopic, percutaneous therapy and liver transplantation (Mousavere et al., 2023).

PSC is characterized by inflammation, fibrosis, and abnormal narrowing and constriction of medium and large ducts in the intrahepatic and/or extrahepatic biliary tree (Cazzagon et al., 2024). The disease targets the cholangiocytes, epithelial cells that line the bile ducts and that play a crucial role in bile production, modification and transport. Cholangiocytes are also involved in liver repair and can become reactive in response to various stimuli, contributing to liver disease. As a result, in PSC livers, bile ducts undergo ductal reactions as a result of inflammation and scarring (Sarcognato et al., 2021; Venkatesh et al., 2022). This leads to the formation of fibrous tissue around the bile ducts as well as loss of ducts, which increases as the condition progresses, although some areas of liver remain relatively normal (Ponsioen et al., 2021).

Cholangiocytes are a source of the vasoactive peptide endothelin-1 (ET-1). Following experimental bile duct ligation, ET-1 is released, inducing vasoconstriction of smooth muscle cells as well as inflammation. These effects are predominantly mediated via activation of ET_A_ receptors. There is evidence that dysregulation of endothelin signalling can contribute to liver pathophysiology. For example, in animal models, ET_A_ blockade beneficially reduces liver fibrosis in bile ducts after ligation (Cho et al., 2000). In ET_B_ knockout mice, sinusoids were reduced in both number and diameter compared to controls, suggesting endothelial ET_B_ blockade may cause sinusoidal constrictions (Ling et al., 2012). In the perfused rat liver, ET-1 induces cholestasis, by increasing portal pressure (Bluhm et al., 1993; Isales et al., 1993). Of particular relevance to the current study expression of genes encoding ET-1 (*EDN1*) and ET_A_ receptor (*EDNRA*) were significantly increased in cholangiocytes isolated from patients with PSC and levels of ET-1 immunoreactivity were elevated in bile and cholangiocyte supernatants compared to controls. These results were replicated in a mouse model of PSC, where crucially the ET antagonist ambrisentan, that has some selectivity for the ET_A_ receptor, reduced ductular reaction, inflammation, fibrosis and angiogenesis (Owen et al., 2023).

A new therapeutic strategy is emerging to target the ET pathway (Ambery et al., 2024), by combining ET_A_ selective antagonists, such as zibotentan, with the sodium-glucose cotransporter 2 protein (SGLT2) inhibitor dapagliflozin, to reduce the fluid retention associated with ET antagonists. This drug combination is being evaluated in a clinical trial in patients with liver cirrhosis with portal hypertension (ZEAL, NCT05516498, Ambery et al., 2024). The rationale for targeting portal hypertension (caused by a range of conditions including PSC) with ET antagonists is supported by the gene encoding ET-1 being identified, by differential gene analysis, as the only significant vasoconstrictor upregulated in portal veins from patients with cirrhosis (Owen et al., 2024). SGLT2 is highly expressed by epithelial cells of the renal proximal convoluted tubules and co-localized with ET_A_ receptors (Williams et al., 2024a). In the kidney, SGLT2 inhibitors prevent the reabsorption of glucose and sodium back into the bloodstream, resulting in their excretion, and thereby reducing fluid retention. Monotherapy using SGLT2 inhibitors have been suggested as potential therapeutic agents for treating NAFLD/NASH by reducing insulin resistance and improving glucose control, lowering hepatic fat accumulation and inflammation (Kaur et al., 2023).

Accumulating evidence from pre-clinical studies, including the mouse model of PSC (Owen et al., 2023), suggests a role for the ET signalling pathway in this condition. Our aim was to compare the cellular localization of the targets for combination therapy, ET receptors and SGLT2, in livers obtained from patients at the time of transplantation to evaluate the potential for this therapeutic strategy in PSC.

## 2 Materials and methods

### 2.1 Materials

All chemicals and reagents used in this study were purchased from Merck (formerly Sigma Aldrich), unless indicated otherwise. Primary and secondary antibodies used in immunohistochemistry experiments in this study are outlined in Supplementary Table S1.

### 2.2 Ethics

Surgical samples were obtained with ethical approval (REC reference 10/H0305/33, 05/Q104/142) and informed consent at the time of liver transplantation from 6 patients with PSC (5 males, 1 female, mean age = 52 years) and from 5 healthy donor livers (3 males, 2 females, mean age = 65 years).

### 2.3 Immunocytochemistry

Immunocytochemistry was performed to localise SGLT2, ET_A_ and ET_B_ receptors in fresh frozen cryostat sections (10 μm, prepared and stored at −70 °C) of surgical samples of human tissue (n = 3/6 individuals per experiment) as previously described (Williams et al., 2024a; Williams et al., 2024b).

Tissue sections were thawed and rehydrated in PBS, fixed for 3–5 min in paraformaldehyde solution (4% buffered, pH 6.9, 1.00496.8350; Sigma-Aldrich) and then washed 3 × 5 min with PBS. Non-specific staining was blocked by incubating with PBS containing 10% donkey sera, for 2 h at room temperature. Tissue sections were then incubated overnight at 4 °C with a panel of primary antibodies against SGLT2 (mouse anti-SGLT2, Abcam; ab58298; amino acid sequences 228-278, diluted at 1:300); ET_A_ (rabbit anti-ET_A_ AER_001; Alomone; diluted at 1:100, against the sequence 413-426, giving a single band in Western blot) and ET_B_ (rabbit anti-ET_B_, Rb D51B; in-house; sequence 428-442, diluted at 1:50, and previously validated by absence of staining in endothelial cell-specific ET_B_ knockout mice (Kelland et al., 2010)). Controls comprised adjacent tissue sections treated with buffer in which primary antisera were omitted. All primary and secondary antibodies were prepared in a diluent consisting of PBS with 3% donkey serum, 0.1% Tween-20, and 3.3 mg/mL bovine serum albumin. Buffer only controls, omitting the primary antibodies, were incubated with diluent alone. All slides were subsequently washed 3x with PBS with 0.1% Tween-20 (PBS/T) before incubation for 1 h at room temperature in the dark with fluorescently conjugated secondary antisera: polyclonal donkey anti-rabbit IgG H&L antibody conjugated to Alexa Fluor 488 (ab150061; Abcam; 1:200); donkey anti-mouse IgG H&L antibody conjugated to Alexa Fluor 555 (ab150110; Abcam; 1:200) prepared in diluent as above. Slides were again washed 3x with PBS/T before incubation with Hoechst 33342 nuclear stain (H3570; Invitrogen) prepared at 10 μg/mL in diluent as above, for 20 min at room temperature in the dark. Following a final 3x washes with PBS/T, slides were blotted dry with lint-free tissue, mounted with ProLong Gold Antifade Mountant, covered with a cover slip, and left at room temperature in the dark to cure (≥48 h).

### 2.4 Multispectral fluorescent high content imaging and whole slide digitisation

Automated fluorescent images (16 bit, 0.325 × 0.325 μm scaling per pixel) of the two antibodies visualised by fluorescent secondary antisera together with Hoechst nuclear stain to visualise all cells present in tissue sections were acquired using an Axio Scan Z1 (Zeiss United Kingdom) slide scanner and digitized using a Hamamatsu Orca Flash camera, equipped with a Plan-Apochromat 20x/NA0.8 M27 objective lens, permitting detection of fluorescence and LED light sources to minimise autofluorescent signal. This automated microscope system allows multiple slides to be imaged using identical settings, in the predetermined profile, without operator intervention. The camera and maximally corrected optics were designed to achieve optimal image quality, the UV-free LED light guarantees a low and consistent illumination source for minimal fading, ensuring reproducible quantitative data.

An initial brightfield scan was used to visualise tissue on slides, and a spline contour tool outlined the tissue to minimise the total region imaged. A profile with three fluorescent channels was used; a first channel (blue), with an LED-Module 385 nm light source set at 10% intensity and 10 ms exposure time at a depth of focus of 1.45 μm for Hoechst 33342 nuclear marker (405 nm wavelength); a second channel (green), with an LED-Module 475 nm light source set at 80% intensity and 30 ms exposure time at a depth of focus of 1.64 μm for 488 nm wavelengths; and a third channel (gold), an LED-Module 567 nm light source set at 80% intensity and 30 ms exposure time at a depth of focus of 1.88 μm for 555 nm wavelengths. Short exposure times were used to both reduce bleaching of fluorophores and minimise autofluorescence to improve signal-to-noise. The whole image of the slide is captured automatically, without operator intervention. All slides used in the study were loaded into the slide scanner and imaged at the same time, using identical settings and using the same predetermined profile. Focal depths were determined by the slide scanner’s in-built Z stacking autofocusing.

### 2.5 Image analysis

All acquired images comprising the entire region defined by the spline contour on the microscope slide, were saved and visualised using ZEN software (Zeiss United Kingdom). For each positive section, an adjacent control section where the primary antisera were omitted was imaged to measure background fluorescence intensity for each of the three separate channels. These settings were retained to capture images for figures, showing positive immunofluorescence above the background. The spline contour was used to draw regions of interest and intensity values were measured in both the positive and equivalent sections in the control tissues (five measurements per region in each tissue replicate). Results of regions showing significant positive immunofluorescence values (grayscales) above background are shown in Supplementary Figure S1 for SGLT2, ET_A_ and ET_B_ antibodies (n = 5 ± standard deviation for controls deviation, n = 3 ± standard deviation in PSC livers, p < 0.05, unpaired, 2-tailed t-test). Relative levels of staining for different protein targets such as ET_A_ *versus* ET_B_ were not compared as antibodies used were likely to have different affinities, leading to variable amplification of signal.

## 3 Results

**FIGURE 1:**
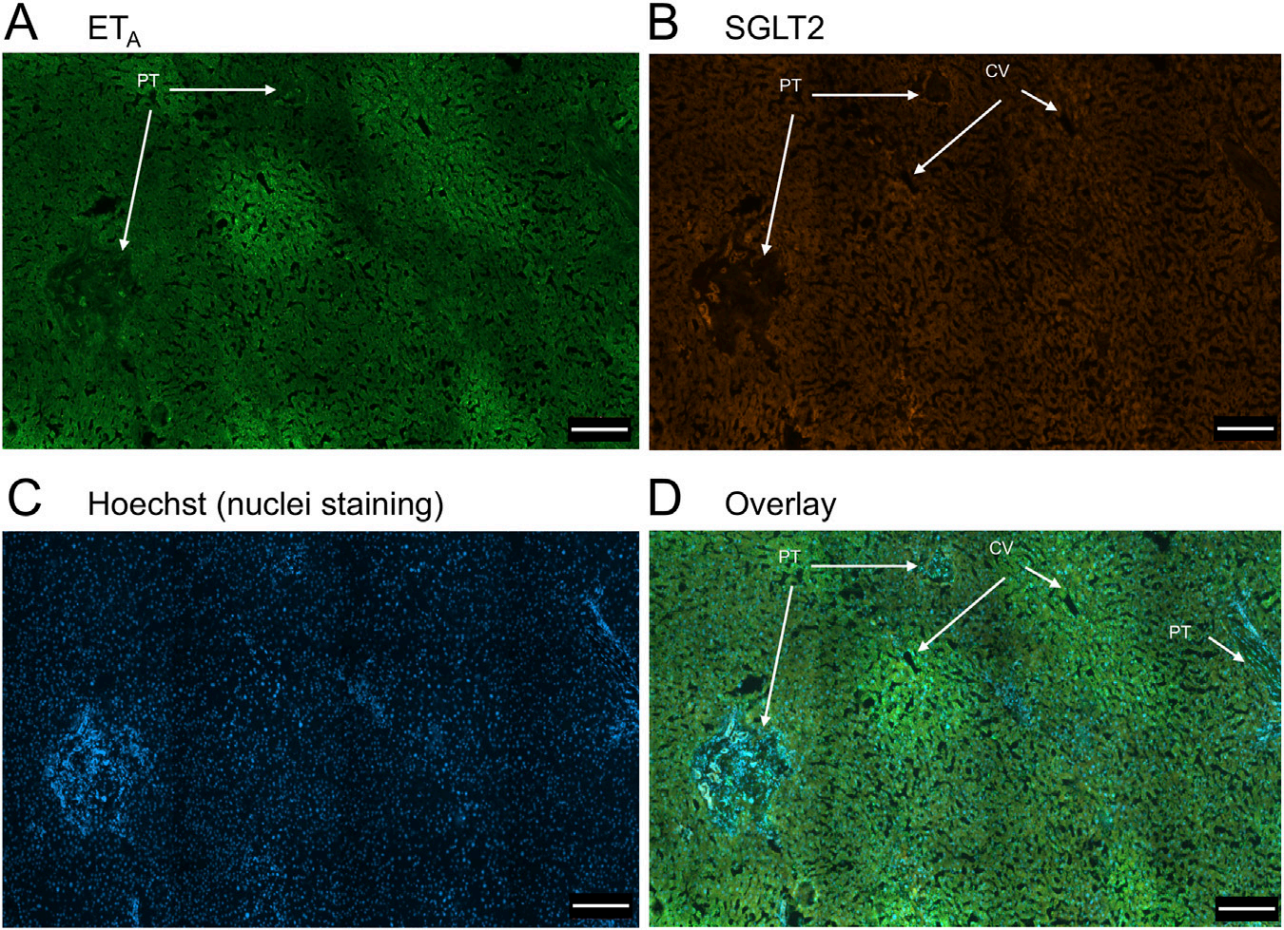
Representative ET_A_ **(A)** and SGLT2 **(B)** immunofluorescence images of sections of normal human liver together with cell nuclei **(C)**. The overlay **(D)** shows co-localization of ET_A_ and SGLT2 to the epithelial cells of bile ducts within the portal tract (PT); SGLT2 was detected in hepatocytes within the hepatic lobules and ET_A_ staining was present in Zone 3, surrounding the central vein (CV). Scale bar = 200 µm.

**FIGURE 2:**
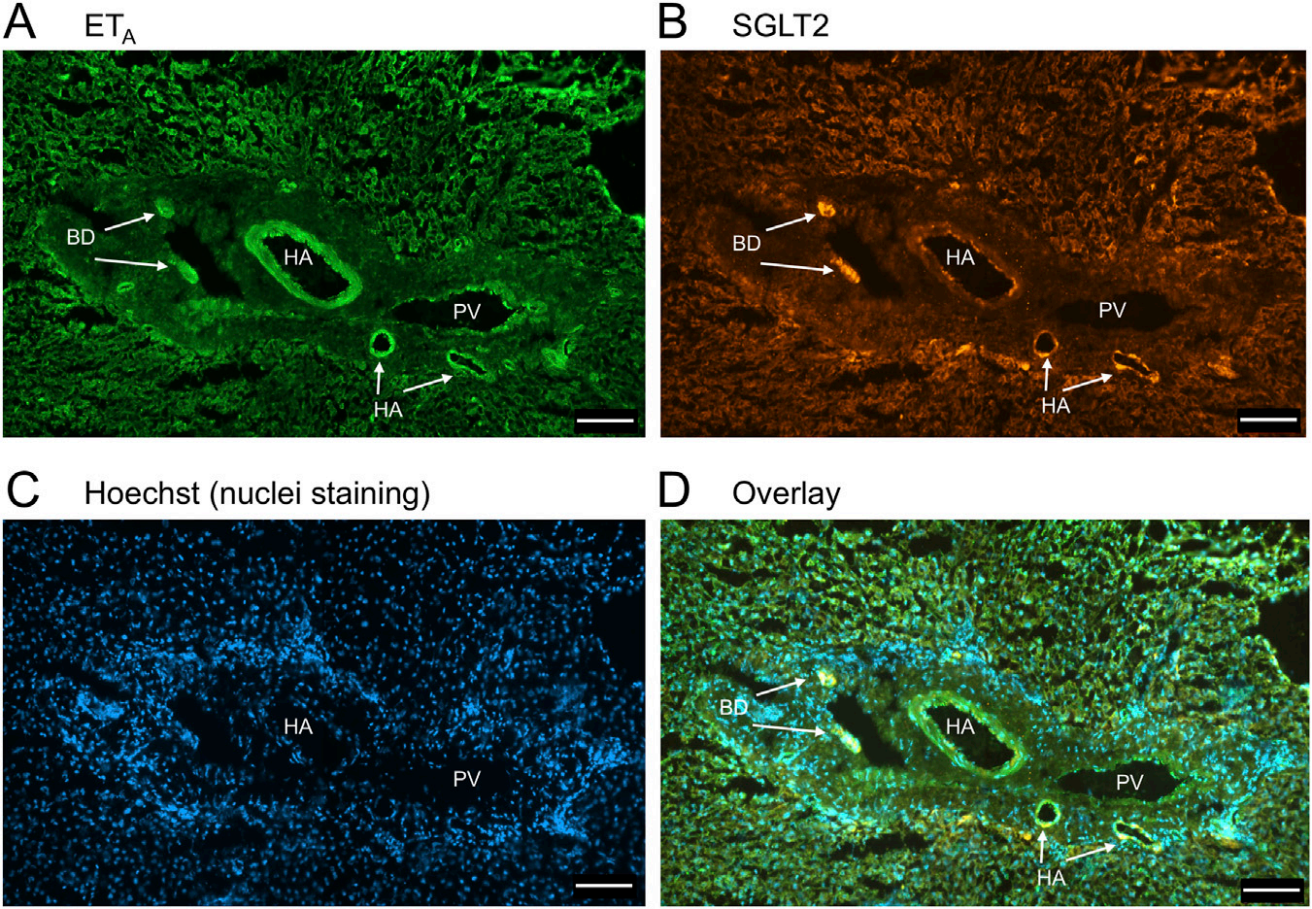
Representative ET_A_ **(A)** and SGLT2 **(B)** immunofluorescence images of sections of normal human liver together with cell nuclei **(C)** within the portal tract. The overlay **(D)** shows co-localization of ET_A_ and SGLT2 at higher magnification to the epithelial cells of bile ducts (BD) and surrounding hepatocytes. Hepatic artery (HA) and portal vein (PV). Scale bar = 100 µm.

**FIGURE 3:**
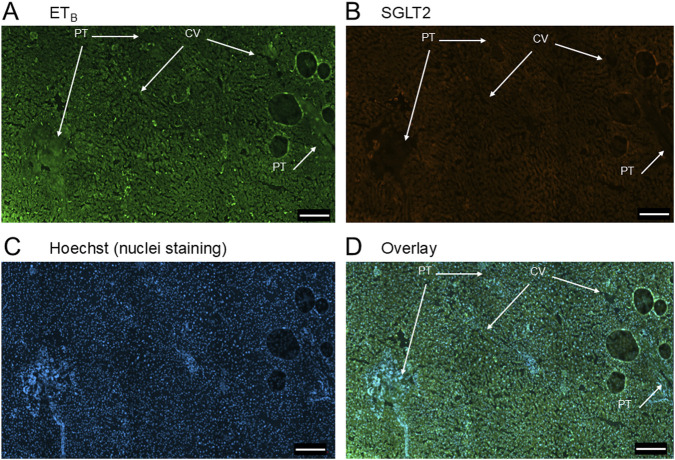
Representative ET_B_ **(A)** and SGLT2 **(B)** immunofluorescence images of sections of normal human liver together with cell nuclei **(C)**. The overlay **(D)** shows co-localization of ET_B_ and SGLT2 to the epithelial cells of bile ducts within the portal tract (PT). SGLT2 was detected in hepatocytes together with ET_B_ staining. Central vein (CV). Scale bar = 200 µm.

**FIGURE 4:**
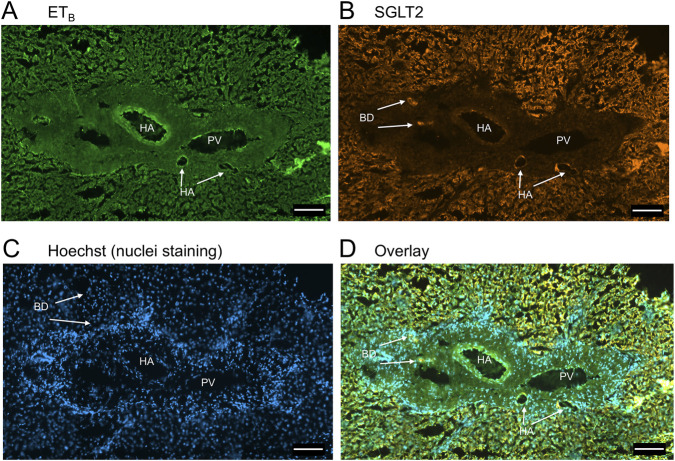
Representative ET_B_ **(A)** and SGLT2 **(B)** immunofluorescence images of sections of normal human liver together with cell nuclei **(C)** within the portal tract. The overlay **(D)** shows co-localization of ET_B_ and SGLT2 at higher magnification to the epithelial cells of bile ducts (BD) and surrounding hepatocytes. Hepatic artery (HA) and portal vein (PV). Scale bar = 100 µm.

In sections of healthy normal human liver (Figure 1), ET_A_ immunofluorescence localised, as expected, to the epithelial cells of bile ducts within the portal tract and to smooth muscle of the central vein, with low levels detectable in hepatocytes forming Zone 3. SGLT2 immunofluorescence was also detectable, primarily on the apical domain of the epithelial cells of bile ducts as well as on hepatocytes at lower but detectable levels above background (autofluorescence visualised in an adjacent section where primary antisera had been omitted). In large hepatic artery and portal vein, ET_A_ immunofluorescence also co-localised, with smooth muscle cells, identified by alpha-actin staining (Supplementary Figures S2, S4). Figure 2 shows an example of the co-localization of both SGLT2 and ET_A_ immunofluorescence at higher magnification in the portal tract. ET_B_ immunoreactivity (Figures 3, 4) localised to hepatocytes but with no zonal distribution and to endothelial cells of central vein. ET_B_ immunofluorescence co-localised, as expected, with endothelial cells lining the blood vessels identified by vWF staining in large arteries and veins as well as lower levels in smooth muscle (Supplementary Figures S3, S5).

**FIGURE 5:**
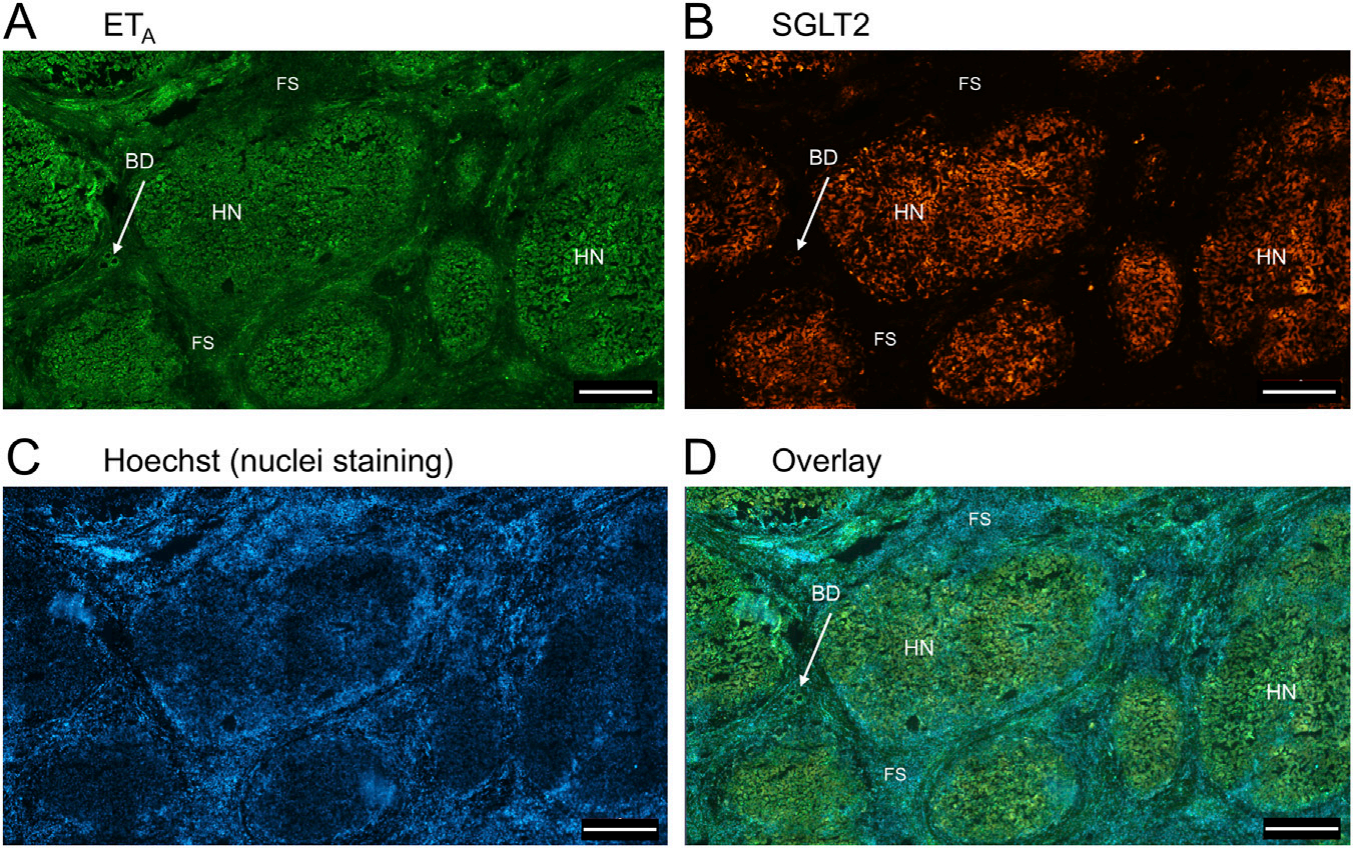
Representative ET_A_ **(A)** and SGLT2 **(B)** immunofluorescence images of in a section of PSC liver together with cell nuclei **(C)**, showing fibrous septa (FS) and nodules of hepatocytes (HN) consistent with cirrhosis. In the overlay **(D)**, SGLT2 and ET_A_ immunofluorescence localize to hepatocytes in nodules (HN) and bile ducts (BD), fibroblasts and blood vessels within the fibrous septa (FS). Scale bar = 500 µm.

**FIGURE 6:**
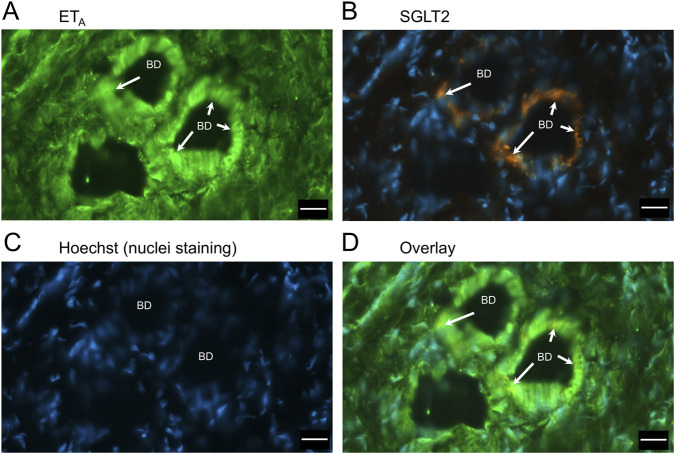
Representative ET_A_ **(A)** and SGLT2 **(B)** immunofluorescence images at higher magnification showing bile ducts (BD), together with cell nuclei **(C)**. In the overlay **(D)**, SGLT2 and ET_A_ immunofluorescence co-localize to the epithelial cells. Arrows indicate co-localization of ET_A_ and SGLT2. Scale bar = 20 µm.

**FIGURE 7:**
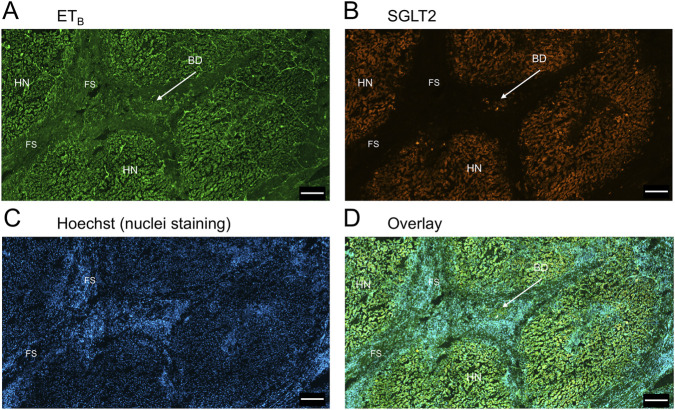
Representative ET_B_ **(A)** and SGLT2 **(B)** immunofluorescence images of in a section of PSC liver together with cell nuclei **(C)**, showing the diseased areas of fibrous septa (FS). In the overlay **(D)**, SGLT2 and ET_B_ immunofluorescence localize to hepatocytes in nodules (HN) and bile ducts (BD), fibroblasts and blood vessels within the fibrous septa (FS). Scale bar = 500 µm.

**FIGURE 8:**
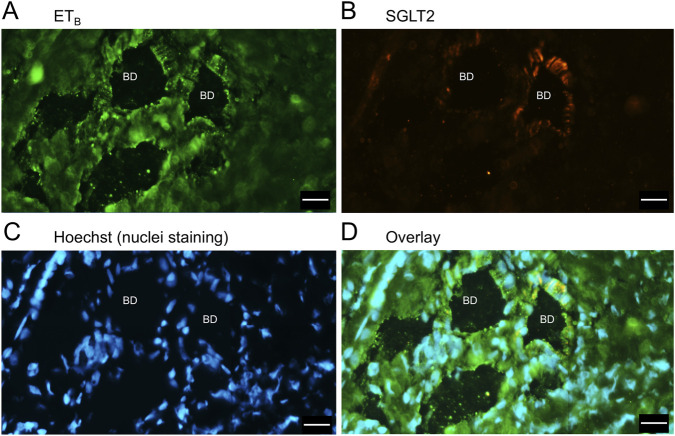
Representative ET_B_ **(A)** and SGLT2 **(B)** immunofluorescence images at higher magnification showing bile ducts (BD), together with cell nuclei **(C)**. In the overlay **(D)**, SGLT2 and ETB immunofluorescence co-localize to the epithelial cells. Scale bar = 20 µm.

In sections of liver from patients with PSC (Figure 5), fibrotic septa (FS) were clearly visualised by the nuclear stain, with low but detectable ET_A_ and SGLT2 immunofluorescence in cells of the fibrous tissue (Supplementary Figure S1). ET_A_ and SGLT2 staining were present in hepatocytes in nodules and bile ducts, with lower levels on microvessels within fibrous septa. Figure 6, at higher magnification, shows ET_A_ and SGLT2 immunoreactivity co-localization to bile duct epithelial cells. Figure 7, shows the same pattern of ET_B_ localised to hepatocytes and bile ducts in nodules, with co-localization to bile duct epithelial cells (Figure 8, also at higher magnification).

**FIGURE 9:**
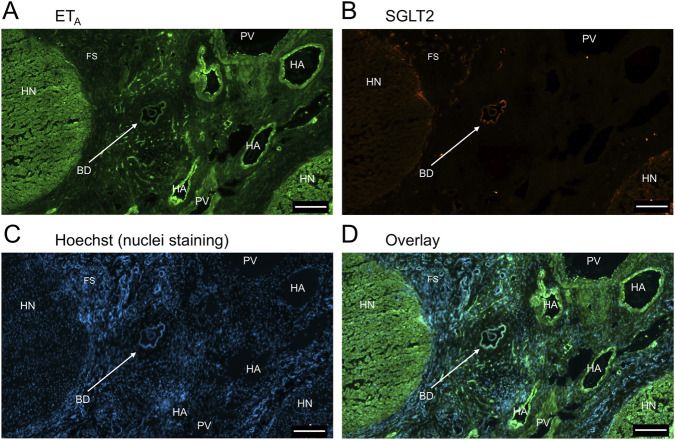
Representative ET_A_ **(A)** and SGLT2 **(B)** immunofluorescence images showing localization to blood vessels in PSC liver together with cell nuclei **(C)**. The overlay **(D)** shows localization of ET_A_ immunofluorescence to smooth muscle of portal veins (PV)and hepatic arteries (HA) within the fibrotic septum (FS), as well as to co-localization with SGLT2 to bile ducts (BD) Hepatocyte nodule (HN). Scale bar = 200 µm.

**FIGURE 10:**
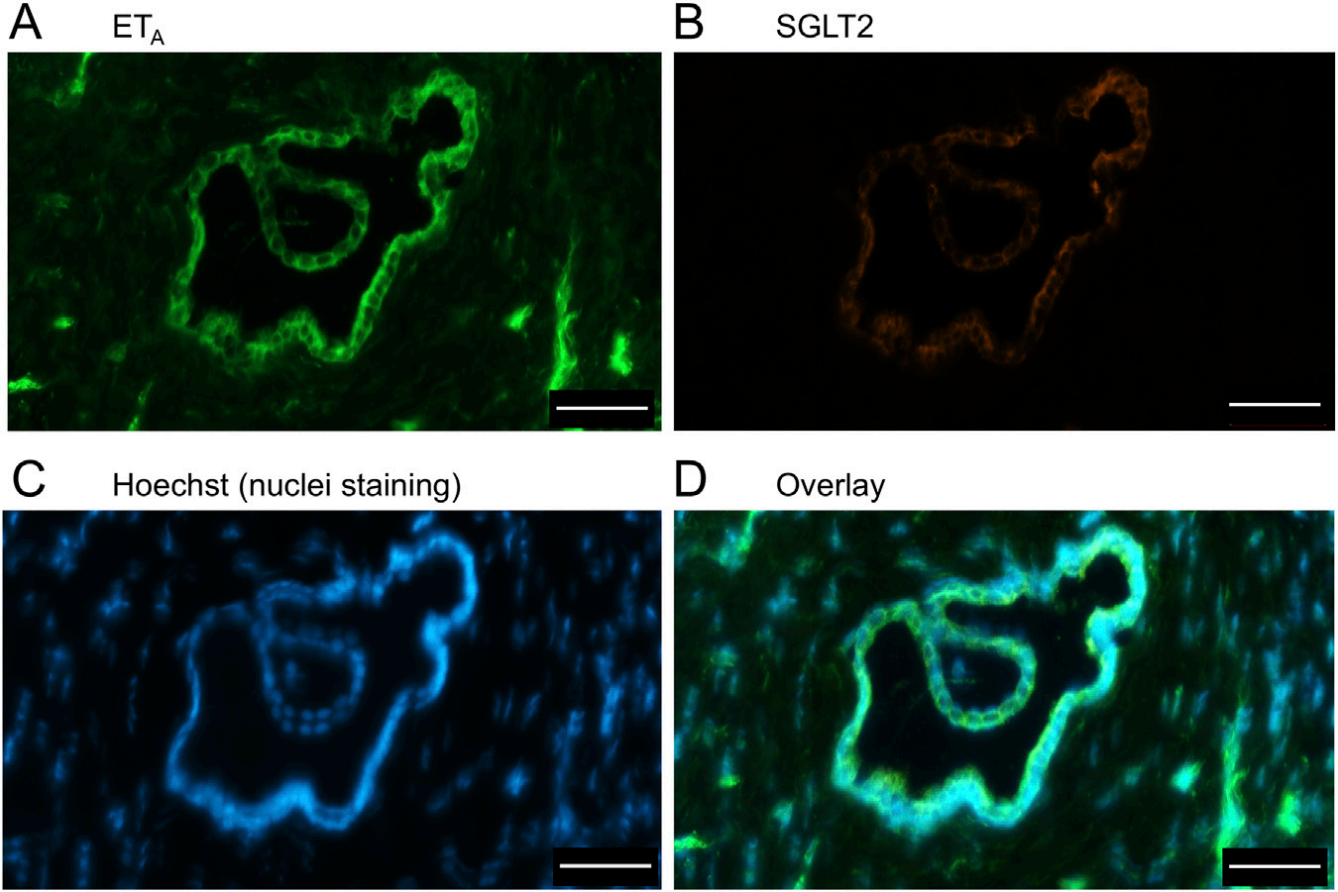
ET_A_ **(A)** and SGLT2 **(B)** immunofluorescence images, together with cell nuclei **(C)**, at higher magnification showing co-localization primarily to the apical domain of bile duct epithelial cells **(D)**. Scale bar = 50 µm.

**FIGURE 11:**
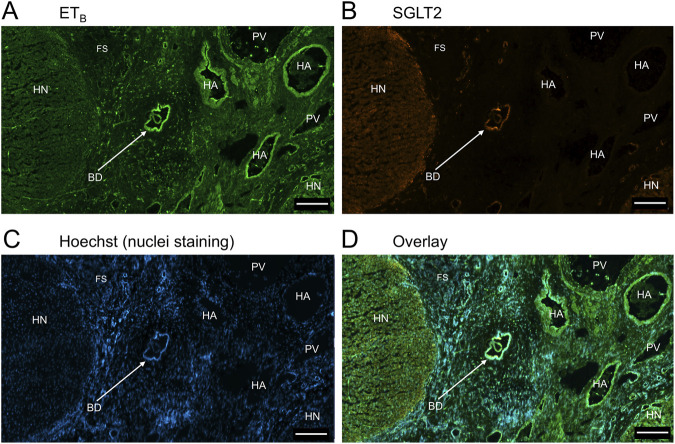
Representative ET_B_ **(A)** and SGLT2 **(B)** immunofluorescence images showing localization to blood vessels in PSC liver together with cell nuclei **(C)**. The overlay **(D)** shows localization of ET_A_ immunofluorescence to smooth muscle of portal veins (PV)and hepatic arteries (HA) within the fibrotic septum (FS), as well as to co-localization with SGLT2 to bile ducts (BD) Hepatocyte nodule (HN). Scale bar = 200 µm.

**FIGURE 12:**
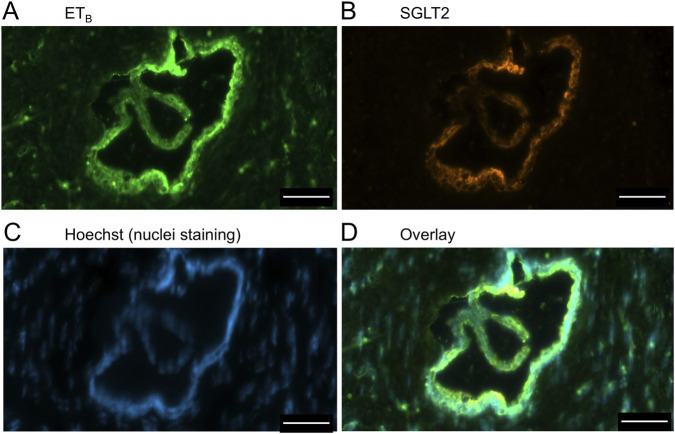
ET_B_ **(A)** and SGLT2 **(B)** immunofluorescence images, together with cell nuclei **(C)**, at higher magnification showing co-localization primarily to the apical domain of bile duct epithelial cells **(D)**. Scale bar = 50 µm.

**FIGURE 13:**
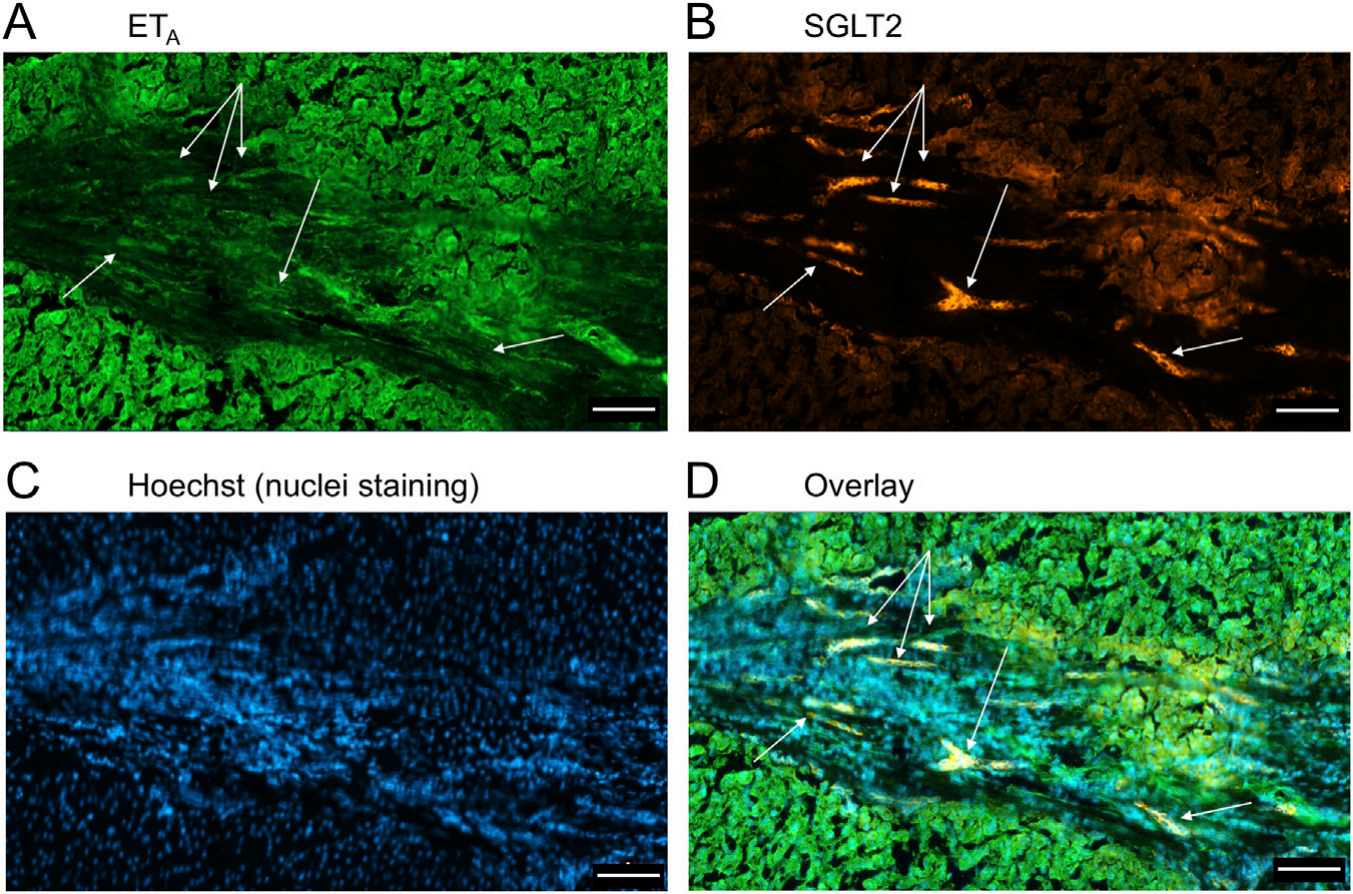
Examples of ET_A_ **(A)** and SGLT2 **(B)** immunofluorescence, together with cell nuclei **(C)**, with co-localization **(D)** in ductular reactions (arrows) in PSC liver tissue. Scale bar = 100 µm.

**FIGURE 14:**
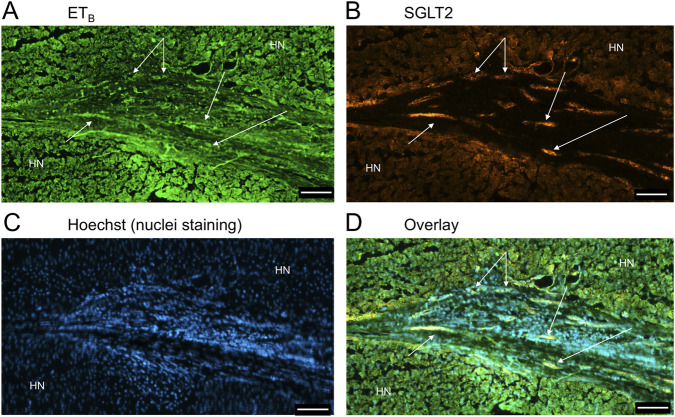
ET_B_ **(A)** and SGLT2 **(B)** immunofluorescence, together with cell nuclei **(C)**, with co-localization **(D)** in ductular reactions (arrows) in PSC liver. Scale bar = 100 µm.

**FIGURE 15:**
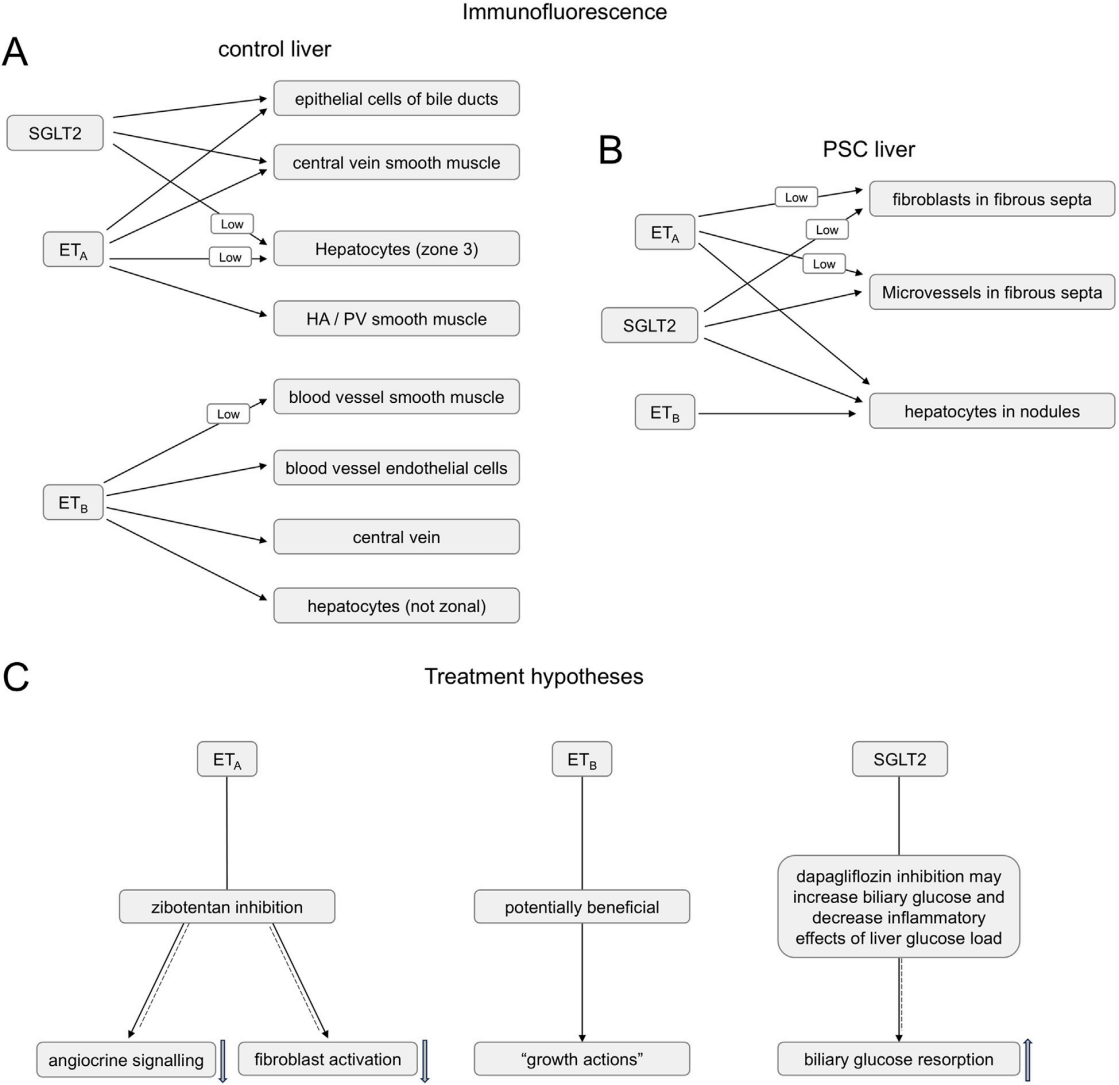
Summary of ET_A_, ET_B_ and SGLT2 immunofluorescence co-localization in control **(A)** and PSC **(B)** livers **(C)**. Summary of hypotheses for the action of ET_A_ antagonist zibotentan blocking angiocrine signalling and fibroblast activation (solid-dashed arrow) and SGLT2 inhibitor dapagliflozin bloking biliary glucose reabsorption (solid-dashed arrow) in PSC liver disease patients, while maintaining beneficial action of ET-1 activating the ET_B_ pathway (solid arrow).

Figure 9 is a representative example of a fibrotic septum encompassing portal vessels, with ET_A_ immunofluorescence localized to smooth muscle of the portal vein and hepatic artery. SGLT2 immunofluorescence was not detected above background in these vessels. Both ET receptor sub-types co-localised to the hepatocyte nodule and bile duct as in healthy liver (Figure 4). The bile duct at higher magnification (Figure 10) shows ET_A_ and SGLT2 immunofluorescence is mainly localised to the apical membrane of the epithelial cells, a similar sub-cellular expression of SGLT2 to that in epithelial cells in the kidney. A similar pattern of ET_B_ is shown in corresponding adjacent sections (Figures 11, 12). Further examples of bile ducts are shown in Supplementary Figures S6, S7. Ductular reactions, that are associated with proliferating cells and a response to injury, reflecting the underlying chronic damage and inflammation characteristic of PSC, are shown in Figures 13, 14, with both receptor subtypes, ET_A_ and ET_B_, co-localising with SGLT2 to epithelial cells. The results for the immunofluorescence localization in control *versus* PSC liver are summarised in Figures 15A,B.

## 4 Discussion

This study has localised targets for ET receptor antagonists and SGLT2 inhibitors in livers from patients with PSC, where there is currently no drug treatment to slow or reverse the damage caused by the disease and there is an unmet need for new treatments (Venkatesh et al., 2022; Bowlus et al., 2023). High blood pressure in the portal vein is the final common pathway in liver cirrhosis. As a consequence, the liver attempts to repair itself, resulting in a distorted architecture related to bands of fibrosis and regenerative nodules of hepatocytes, regardless of aetiology (Bosch et al., 2015). This includes conditions that cause inflammation in the bile ducts such as PSC. ET antagonists are thought to be beneficial by controlling portal hypertension, lowering intrahepatic vascular resistance and hepatic microcirculation resistance, and reducing risks such as variceal bleeding. Although it is rare for patients with PSC to get significant fluid overload, except at the very end of their disease, SGLT2 inhibitors not only lower systemic blood pressure but importantly also reduce fluid retention which can be a significant side effect with ET antagonists. This was the rationale for co-administering both zibotentan and dapagliflozin in patients with portal hypertension in a Phase 2 clinical study (ZEAL, NCT05516498, Ambery et al., 2024).

### 4.1 ET receptor expression in liver from PSC patients

In healthy human vessels, including arteries, veins, and micro-vessels, ET_A_ receptors, measured by ligand binding and visualised by immunocytochemistry, predominate on smooth muscle, compared with ET_B_. In these vessels ET-1 mediates vasoconstriction that can be fully reversed by ET_A_ selective antagonists. In contrast, ET_B_ receptors are localised on endothelial cells, indirectly mediating vasodilation via release of endothelium derived factors such as nitric oxide (Davenport et al., 2016). The results of this present study show a similar pattern of receptor expression is retained in the PSC vasculature, consistent with the hypothesis that ET antagonists may be beneficial in controlling portal hypertension in these individuals. In patients with other conditions, such as Type 2 diabetes (T2DM), monotherapy with SGLT2 inhibitors, albeit by acting via targets external to the liver (on kidney epithelial cells of proximal convoluted tubules), also contributes to modest lowering of blood pressure promoting diuresis and natriuresis thus reducing fluid volume. Whilst usefully reducing blood pressure, the highly selective ET_A_ antagonist zibotentan, reduced glycated haemoglobin and low-density lipoprotein cholesterol in addition to any effect produced by concomitant statins (Morrow et al., 2024) that may provide additional clinical benefit. This is relevant, as elevated LDL levels are common in PSC patients and can contribute to cardiovascular risks, especially if they have hypercholesterolemia due to biliary obstruction.

In PSC liver, both ET_A_ and ET_B_ immunofluorescence was localised to groups of fibroblasts within the fibrous septa. These bands of scar tissue can restrict hepatic blood flow and, if severe, lead to cirrhosis. Low levels (detectable above background) of SGLT2 immunofluorescence are also present in these cells. ET-1 stimulation of ET_A_ receptors is associated with fibrosis progression, while ET_B_ activation has growth-inhibitory actions. When the liver is injured, hepatic stellate cells (HSCs) can transform into myofibroblasts producing extracellular matrix components, contributing to the development of liver fibrosis. Koda et al. (2006) for example, showed ET-1 dose-dependently stimulated the expression of procollagen mRNA and the strongest profibrogenic cytokine TGFβ-1, in first passage HSCs *in vitro*. The effect of ET-1 was blocked by an ET_A_ but not an ET_B_ antagonist. In contrast, using HSCs from explants of human liver, Mallat et al. (1995) reported ET-1 binding to ET_B_ receptors caused a potent growth inhibition of human myofibroblasts, suggesting an advantageous role in the negative control of liver fibrogenesis. If translated *in vivo*, these results suggest an ET_A_ selective antagonist would have benefit in blocking the detrimental ET-1 stimulated proliferation of myofibroblasts but sparing the beneficial ET_B_ pathway implied by these studies. Our results support the detailed and compelling study by Owen et al., 2023 who demonstrated genes encoding ET-1 and ET_A_ receptor were increased in mouse and human PSC liver sections. ET-1 peptide was increased in isolated mouse cholangiocyte supernatants as well as human bile and cholangiocyte supernatants. Importantly, ET_A_ inhibition by a clinically approved antagonist, reduced ductular reaction, inflammation, fibrosis, and angiogenesis in the PSC mouse model. The authors concluded ET_A_ regulated biliary angiocrine signalling may therefore influence endothelial cells.

### 4.2 PSC and cancer

PSC patients are at an increased risk of developing cancers including cholangiocarcinoma, gallbladder cancer and colorectal carcinoma compared to the general population (Fung et al., 2019; Bowlus et al., 2023) with cancer the cause of death in almost half of PSC cases. Of interest is that ET_A_ antagonists, such as zibotentan, have been tested in clinical trials for efficacy in some cancers although results have been disappointing. For example, a multi-centre, randomized, double-blind, placebo-controlled phase 2 study (FOLFERA) tested the safety and efficacy of irinotecan and 5-fluorouracil and folinic acid in combination with zibotentan in advanced colorectal cancer (Thomas and Casbar, 2013; NCT01205711). Although zibotentan was well tolerated there was no improvement in progression free survival compared to placebo and the study was terminated before recruitment was completed. Similarly, zibotentan progressed to Phase 3 trials for refractory prostate cancer but without demonstrating clinical benefit (NCT 00554229; NCT 00617669; NCT 00626548). Therefore, although there is a large body of research implicating ET pathways in cancer progression (Harrison et al., 2024), for example both ET_A_ and ET_B_ receptors are reportedly up-regulated in a human cholangiocarcinoma cell line compared with normal cholangiocytes (Fava et al., 2009), it is unlikely that the use of ET antagonists will provide additional benefit in reducing the incidence of PSC associated cancers in these patients. Further studies will be required to confirm or refute this. An alternative strategy of decreasing ET-1 availability by either enhancing its breakdown with recombinant neprilysin or inhibiting its synthesis with an endothelin-converting enzyme inhibitor has been shown to reduce tumorigenic traits of gallbladder cancer cells (Vidal-Vidal et al., 2025). What is of relevance to PSC patients is that, whereas some ET antagonists such as the mixed ET_A_/ET_B_ antagonist bosentan are toxic to the liver, analysis of data from pooled Phase 2b and Phase 3 clinical trials in cancer patients showed that the highly ET_A_ selective zibotentan does not produce drug-induced hepatotoxicity following chronic administration (Fettiplace et al., 2024).

### 4.3 Extra-renal expression of SGLT2

Previously, the SGLT2 transporter in humans was thought to be mainly restricted to the renal proximal convoluted tubules, resulting in increased glucose excretion favouring improved glycaemic control in T2DM and diuresis. Recent research using immunocytochemistry suggests the SGLT2 protein is more widely expressed in human tissue. For example, in cardiomyocytes obtained from biopsies (Marfella et al., 2022), and in cardiac tissue sections obtained following transplantation for cardiomyopathy as well as normal tissue, the transporter co-localised with both ET receptor sub-types (Williams et al., 2024a).

In this study, SGLT2 immunofluorescence (together with ET receptors) localised to epithelial cells of bile ducts and hepatocytes in both normal and PSC livers. These results are in agreement with Nakano et al. (2022) who reported a similar expression in biopsies from patients with chronic liver disease (cause unspecified) although they did not examine healthy tissue. Similarly, all three proteins localised to ductal reactions. This reflects the response of the liver to injury, involving the proliferation of bile duct cells (cholangiocytes) to promote beneficial regeneration but also associated with fibrosis and inflammation.

Whether SGLT2 transporters in the liver have a physiological or pathophysiological role remains to be established. Bile duct epithelial cells reabsorb glucose from bile into the blood by an active transport mechanism, principally SGLT1 (sodium-glucose linked transporter 1) and GLUT1 (glucose transporter 1). If SGLT2 has a similar role, an inhibitor may have a beneficial action in lowering the overall glucose load in the liver, reducing inflammation and fibrosis. Similarly in hepatocytes, while the *SLC2A2* gene encoding GLUT2 (glucose transporter 2) is highly expressed and is important for glucose transport and homeostasis, SGLT2 may also have a role and a further potential target for inhibition.

Intriguingly, a retrospective cohort analysis on data from ∼1,900 patients with T2DM over 2.8 years demonstrated that SGLT2 inhibitor treatment correlated with a substantially lower (38%) risk of developing biliary diseases. There was also a downward trend for this risk reduction continuing beyond 2 years (Gao et al., 2024). The authors explored the potential mechanism of action in a mouse model maintained on a lithogenic diet (40% high fat, 1.25% cholesterol and 0.5% cholic acid) where the SGLT2 inhibitor reduced gallstone formation (affecting about 75% of PSC patients). Possible mechanisms of action in this model were reduced liver injury and dyslipidemia that would be beneficial in PSC patients, as well as improved gallbladder motility and bile acid production. The latter actions may help to maintain bile flow thus reducing cholestasis. In agreement with this, one of the treatments for PSC patients, is ursodeoxycholic acid, a secondary bile acid. In conclusion data from our study, combined with emerging results from animal models, suggest a combination of an ET_A_ selective antagonist with an SGLT2 inhibitor acting via distinct pathways (Figure 15C) may be beneficial in PSC patients for whom current treatment options are limited.

## Data Availability

The raw data supporting the conclusions of this article will be made available by the authors, without undue reservation.
