# Supplementary material for: Localization of the therapeutic targets for endothelin receptor antagonists and sodium-glucose co-transporter 2 inhibitors in the chronic liver disease, primary sclerosing cholangitis
Source: Front Pharmacol. 2025 Sep 29;16:1680875. doi: 10.3389/fphar.2025.1680875 (PMC12516093; doi:10.3389/fphar.2025.1680875)
Supplement: Supplementary file 1 [file Table1.docx]

Supplementary information.

Table S1 Antisera

| Antibody (target) | Dilution | Species | Source | Cat. No. | Cites | CiteAb |
| --- | --- | --- | --- | --- | --- | --- |
| Primary Antibody |  |  |  |  |  |  |
| ET_A_ | 1:100 | Rabbit | Alomone | AER_001 |  | <https://www.biocompare.com/9776-Antibodies/1047886-AntiETA/> |
| ET_B_ | 1:50 | Rabbit | In-house | Rb D51B |  |  |
| SGLT2 | 1:300 | mouse | AbCam | ab58298 |  | <https://www.citeab.com/antibodies/780424-ab58298-anti-sglt2-antibody-3g8> |
| Secondary Antibody |  |  |  |  |  |  |
| anti-rabbit IgG AF488 | 1:200 | donkey | abcam | ab150061 | 111 | <https://www.citeab.com/antibodies/2359550-ab150061-donkey-anti-rabbit-igg-h-l-alexa-fluor-48?des=e685c77aadc34a27> |
| anti-mouse  IgG AF555 | 1:200 | donkey | abcam | ab150110 | 44 | <https://www.citeab.com/antibodies/2359605-ab150110-donkey-anti-mouse-igg-h-l-alexa-fluor-555?des=14ca7787136f079e> |
| Additional antibodies |  |  |  |  |  |  |
| smooth muscle α-actin | 1:100 | mouse | Agilent (Dako) | M0851 |  | <https://www.citeab.com/antibodies/2414737-m0851-actin-smooth-muscle-concentrate> |
| von Willebrand Factor | 1:50 | mouse | Agilent (Dako) | M0616 | 170 | <https://www.citeab.com/antibodies/2414842-m0616-von-willebrand-factor-concentrate> |
| Hoechst 33342 | 1:1000 | n/a | Invitrogen | H3570 |  | <https://www.citeab.com/biochemicals/13235282-h3570-hoechst-33342-trihydrochloride-trihydrate> |
