# Supplementary material for: Localization of the therapeutic targets for endothelin receptor antagonists and sodium-glucose co-transporter 2 inhibitors in the chronic liver disease, primary sclerosing cholangitis
Source: Front Pharmacol. 2025 Sep 29;16:1680875. doi: 10.3389/fphar.2025.1680875 (PMC12516093; doi:10.3389/fphar.2025.1680875)
Supplement: Supplementary file 2 [file DataSheet1.docx]

Supplementary information.


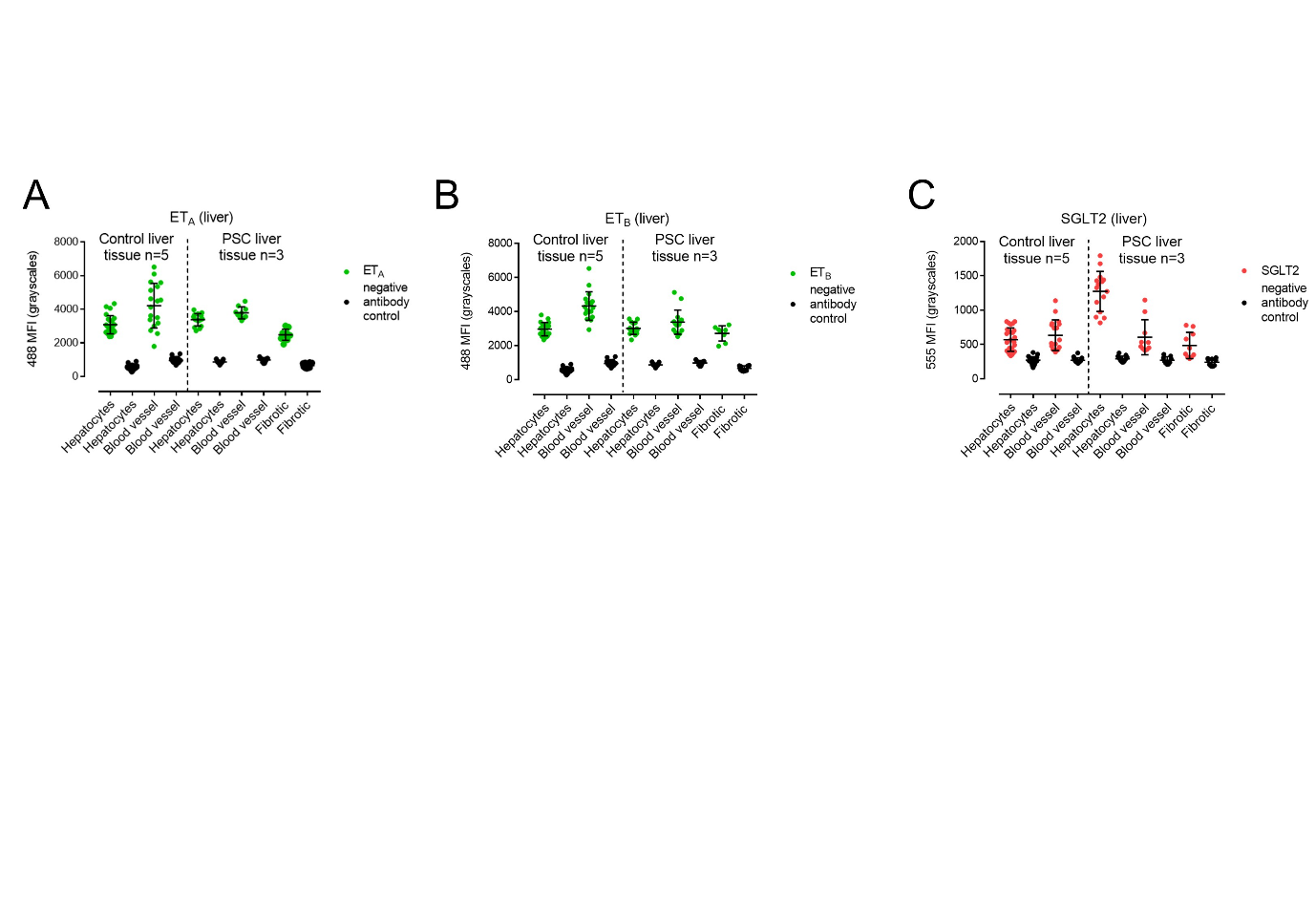


Supplementary Figure S1. Liver tissue regions showing significant (p<0.05) positive immunofluorescence (grayscales) above background for, ET_A_, ET_B_, and SGLT2 antibodies (n=5±standard deviation for controls deviation, n=3±standard deviation in PSC livers). Values were compared to respective negative antibody control using an unpaired, 2-tailed *t*-test.


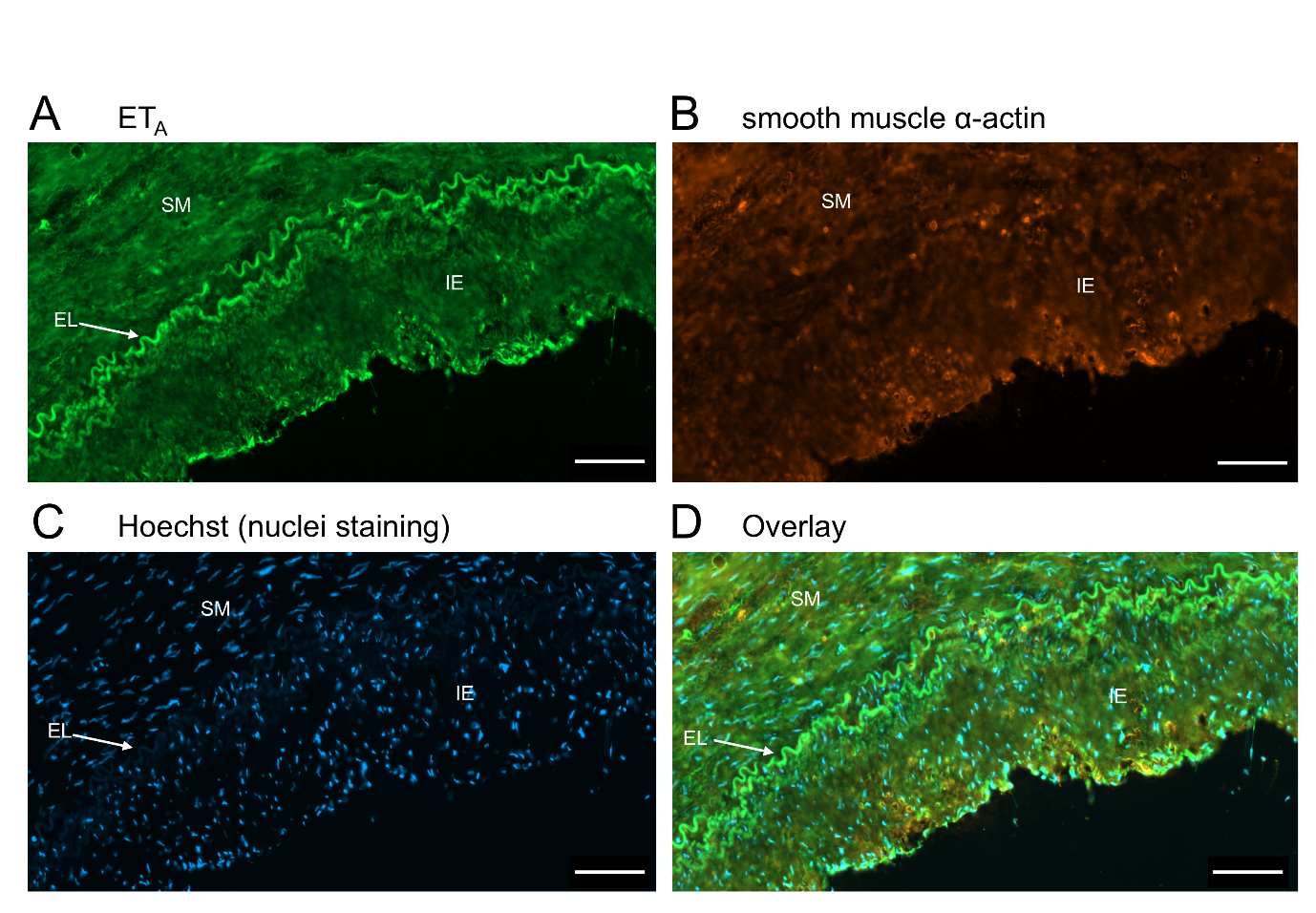


Supplementary Figure S2. ET_A_ (A), α-actin (B) immunofluorescence showing co-localization to smooth muscle (SM) and cells within the intimal expansion (IE) in normal hepatic artery. The elastic laminae (EL) is also visible owing to green autofluorescence. Scale bar = 100µm


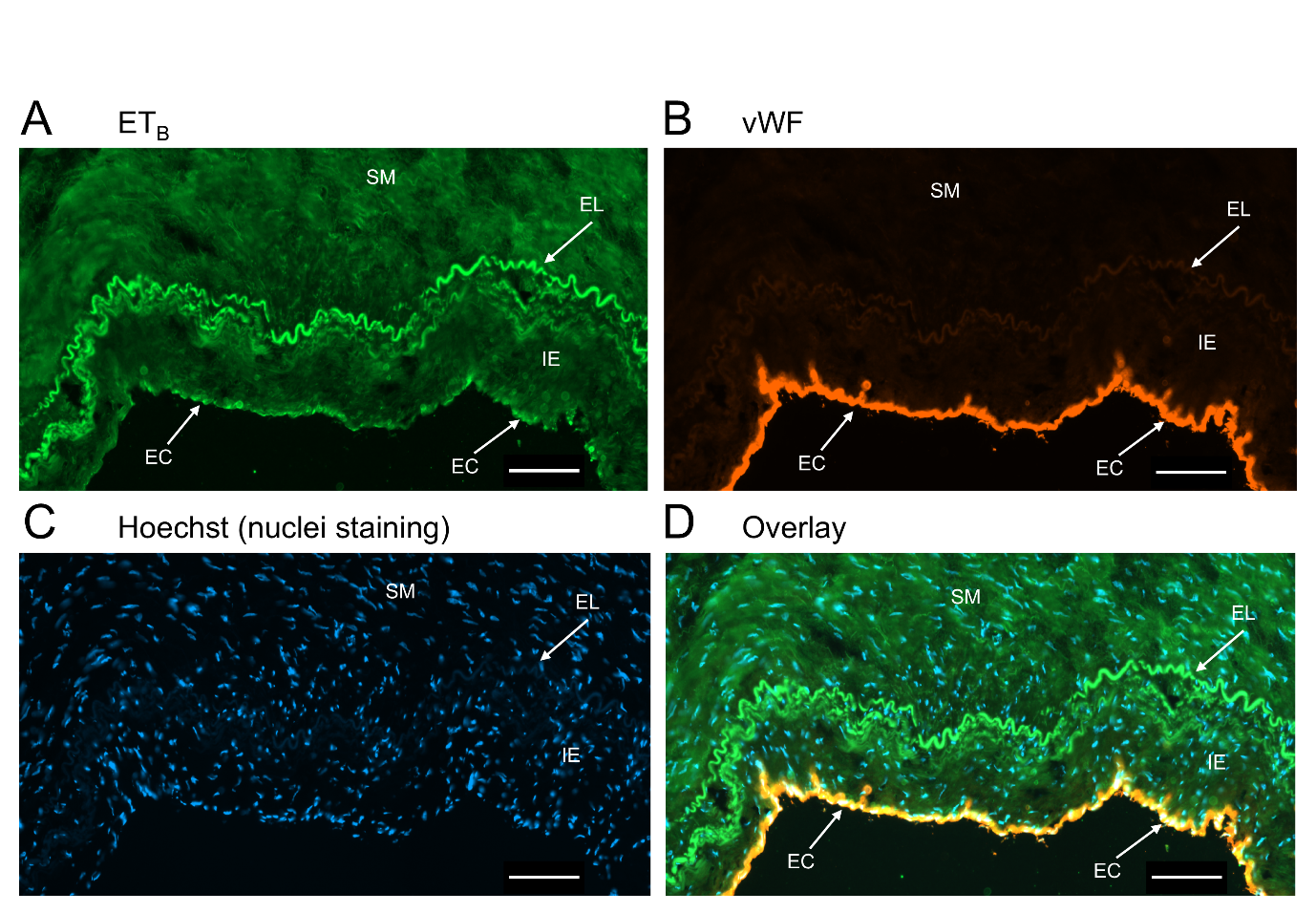


Supplementary Figure S3. ET_B_ (A), vWF (B) immunofluorescence showing co-localization to endothelial cells and smooth muscle (SM) and cells within the intimal expansion (IE) in normal hepatic artery. The elastic laminae (EL) is also visible owing to green autofluorescence. Scale bar = 100µm


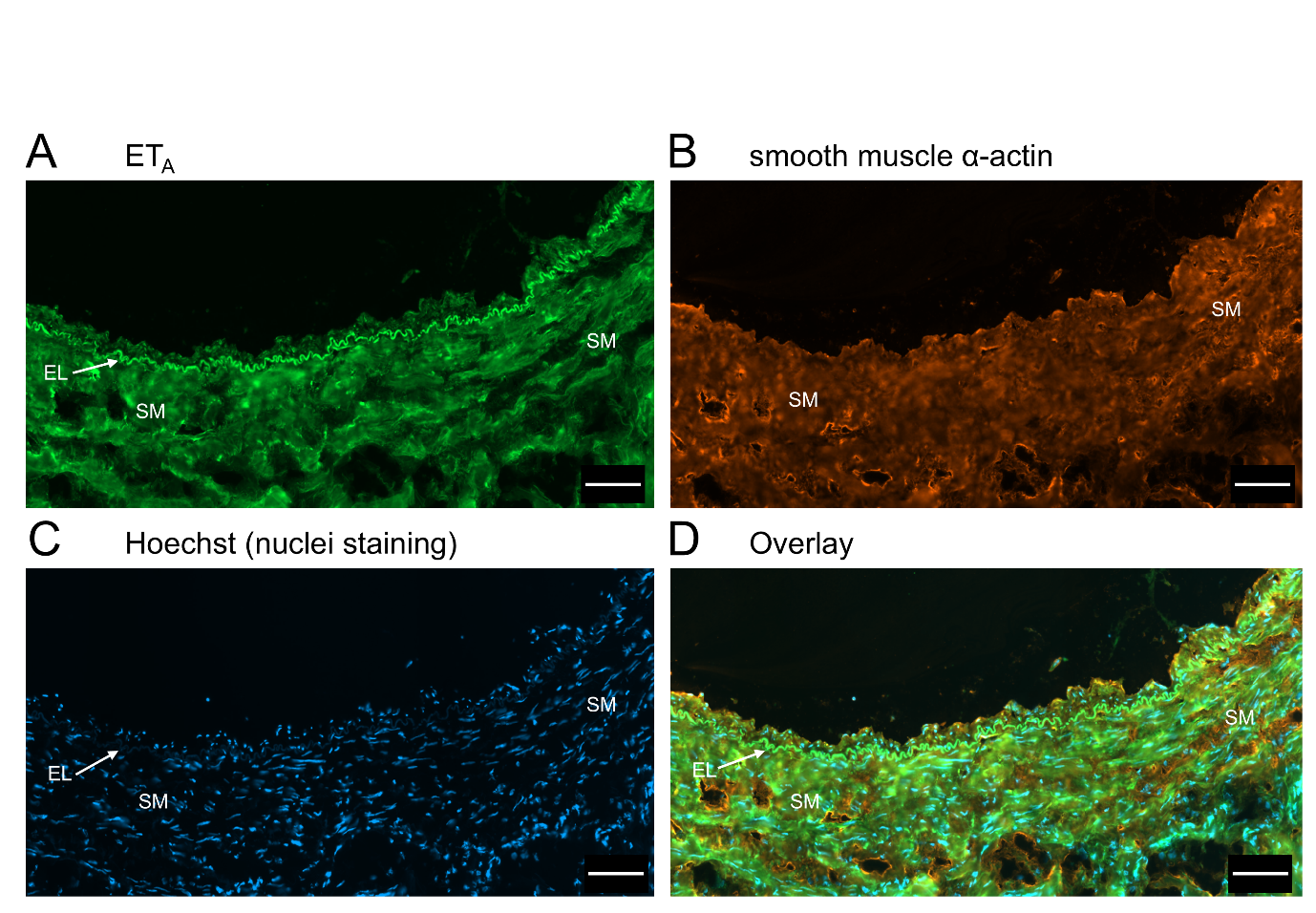


Supplementary Figure S4. ET_A_ (A), α-actin (B) immunofluorescence showing co-localization to smooth muscle (SM) in normal portal vein. The elastic laminae (EL) is also visible owing to green autofluorescence. Scale bar = 100µm


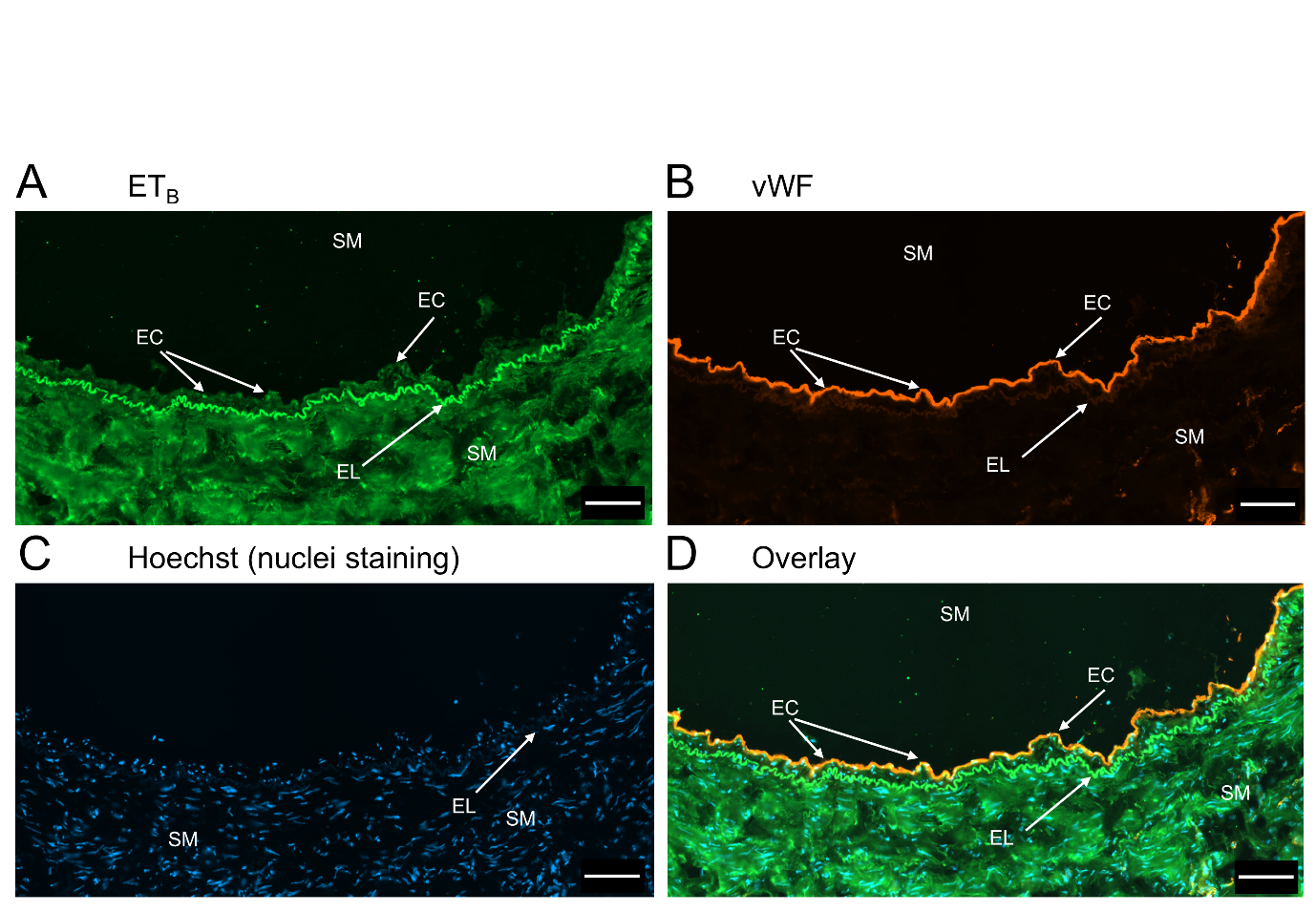


Supplementary Figure S5. ET_B_ (A), vWF (B) immunofluorescence showing co-localization to endothelial cells and smooth muscle (SM) in normal portal vein. The elastic laminae (EL) is also visible owing to green autofluorescence. Scale bar = 100µm


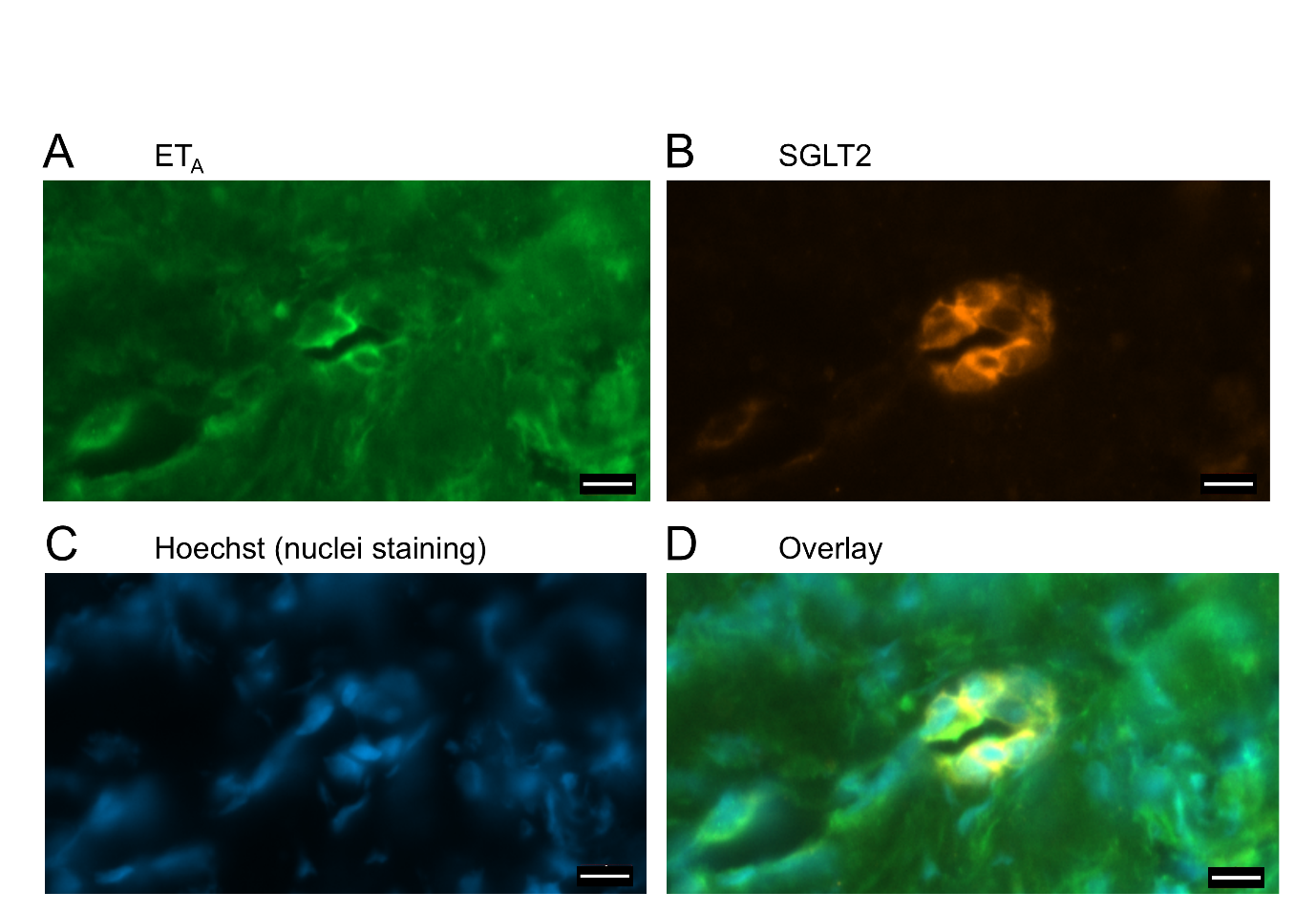


Supplementary Figure S6. SGLT2 and ET_A_ immunofluorescence co-localization in human PSC liver tissue: Bile ducts – small, Scale bar = 10µm


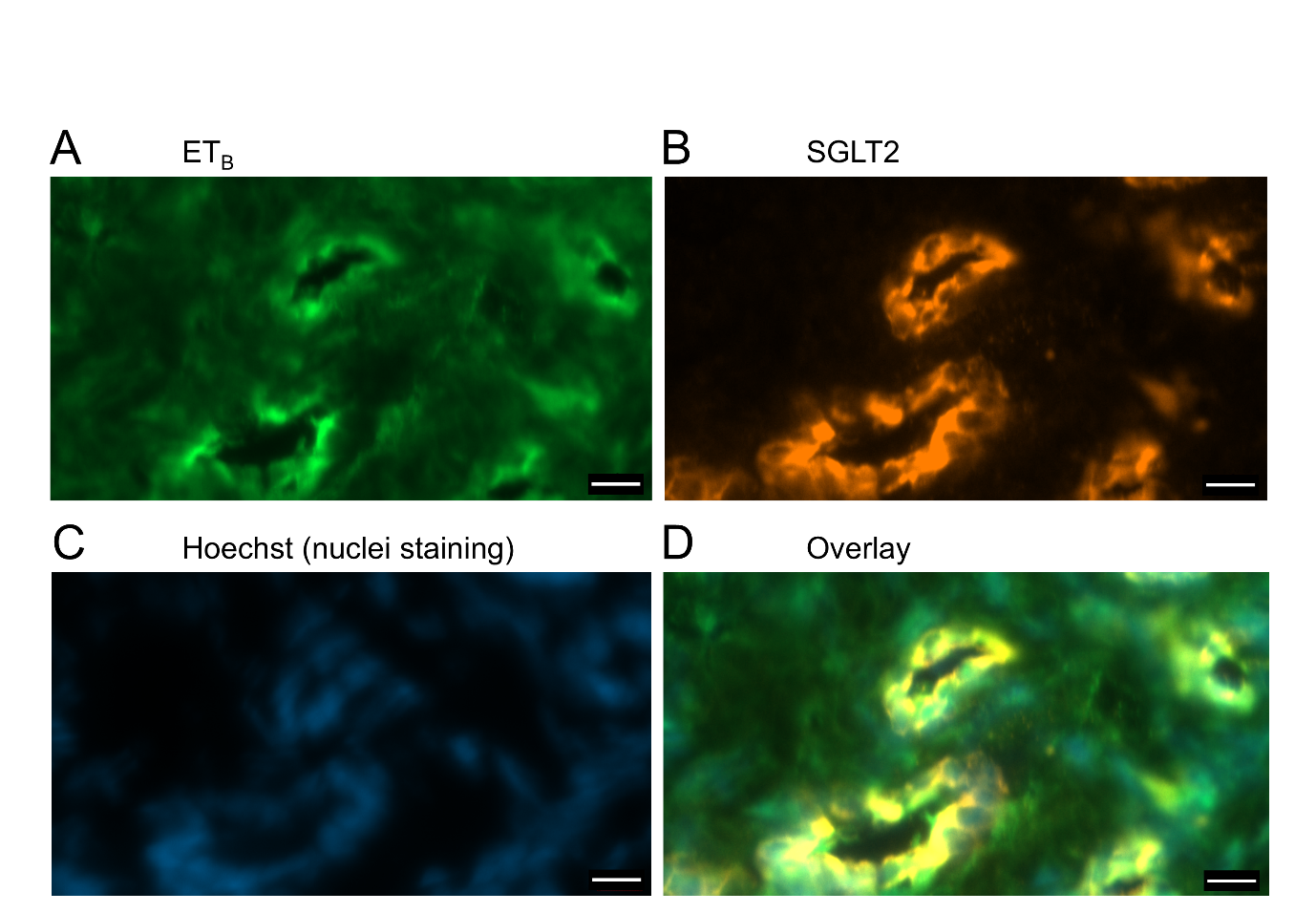


Supplementary Figure S7. SGLT2 and ET_B_ immunofluorescence co-localization in human PSC liver tissue: Bile ducts – small, Scale bar = 10µm
